# Clinical characteristics and outcomes of children admitted to adult intensive care and high-dependency units in Kenya: a multicenter registry-based analysis

**DOI:** 10.3389/fped.2025.1672012

**Published:** 2025-12-05

**Authors:** Wambui Mwangi, Carolyne Njoki, Ronnie Kaddu, Nabukwangwa Simiyu, Demet Sulemanji, Peter Oduor, Dilanthi Gamage Dona, Teddy Thaddeus Abonyo, Patricia Wangeci, Thomas Kabanya, Selina Mutuku, Annastacia Kioko, Peter Mburu Kamau, Eunice Tole, Abigail Beane, Rashan Haniffa, Arjen Dondorp, Marcus J. Schultz, Job van Woensel, David Misango, Luigi Pisani, Wangari Waweru-Siika

**Affiliations:** 1 Department of Anesthesia and Critical Care, Mwai Kibaki Hospital-KNH Annex, Othaya, Kenya; 2 Critical Care Society of Kenya, Nairobi, Kenya; 3 Intensive Care Unit, Aga Khan University Hospital, Nairobi, Kenya; 4 Intensive Care Unit, Aga Khan Mombasa Hospital (AKM), Mombasa, Kenya; 5 Intensive Care Unit, Kisii County and Referral Hospital, Kisii, Kenya; 6 Department of Surgery, Egerton University, Nakuru, Kenya; 7 Nat Intensive Care Surveillance-MORU, Colombo, Sri Lanka; 8 Intensive Care Unit, Nyeri County and Referral Hospital, Nyeri, Kenya; 9 Department of Anesthesia and Intensive Care, MP Shah Hospital, Nairobi, Kenya; 10 Mahidol Oxford Tropical Medicine Research Unit (MORU), Bangkok, Thailand; 11 Nuffield Department of Medicine, University of Oxford, Oxford, United Kingdom; 12 Department of Global Health, Amsterdam UMC, Amsterdam, Netherlands; 13 Amsterdam Institute of Global Health and Development, Amsterdam, Netherlands; 14 Department of Anesthesia, General Intensive Care and Pain Management, Division of Cardiothoracic and Vascular Anesthesia & Critical Care Medicine, Medical University of Vienna, Vienna, Austria; 15 Department of Intensive Care, Amsterdam UMC, Amsterdam, Netherlands; 16 Department of Pediatric Intensive Care, Amsterdam UMC, Amsterdam, Netherlands; 17 Department of Precision-Regenerative Medicine and Jonic Area (DiMePRe-J), Section of Anesthesiology and Intensive Care Medicine, University of Bari “Aldo Moro”, Bari, Italy

**Keywords:** outcome, intensive care unit, children, LMIC (low and middle income countries), Kenya, high dependency unit, mortality

## Abstract

**Problem:**

There is limited information on the prevalence, clinical features and outcomes of pediatric patients admitted to adult intensive care units (ICUs) or high-dependency units (HDUs) in low and middle income countries (LMICs).

**Objective:**

To evaluate the clinical characteristics and outcomes of critically ill children admitted to adult ICU or HDU in Kenya.

**Methods:**

We conducted a registry–enabled study leveraging on data collected progressively in the Kenya Critical Care Registry comprising six ICUs and five HDUs. We included all consecutive encounters of patients younger than 18 years who were admitted to the study units from January 2021 to June 2022.

**Outcomes:**

The primary outcome was ICU or HDU mortality; secondary outcomes included clinical management, duration of invasive ventilation, length of stay in the ICU or HDU and risk factors for mortality.

**Results:**

Of the 5012 ICU and HDU admissions, 466 (9.1%) were patients younger than 18 years. Their median age was 2.0 [0.4–9.0] years, with 173 (37.1%) children being under one year. Medical admissions accounted for 357 (76.6%) cases, while 109 (23.9%) were surgical. Almost half of the children received invasive ventilation, whereas noninvasive ventilation was rarely used. The use of cardiovascular support and renal replacement therapy was also uncommon. Approximately one quarter of children were sedated on admission, and more than 80% received at least one antibiotic. The overall ICU or HDU mortality rate was 34.5%, higher in medical cases than in surgical cases (39.5 vs. 18.3%, *p* < 0.001). Independent risk factors for mortality were age under 28 days, admission due to a medical reason and receiving invasive ventilation.

**Conclusions:**

In a representative sample of Kenyan ICUs, one out of nine admissions to adult ICUs and HDUs involves a child, who often receive invasive ventilation and have a high crude mortality rate. In this cohort of patients, all risk factors for mortality were non-modifiable.

## Introduction

Children in low and middle–income countries (LMICs) bear a significant burden of critical illness due to conditions like sepsis, trauma, nutritional deficiencies, post–surgical complications and neurological disorders (1–3). Despite this, specialized pediatric critical care services remain significantly limited in LMICs across Africa. In Kenya, critically ill children are frequently admitted to intensive care units (ICUs) and high-dependency units (HDUs) that are primarily, or exclusively, designed for adult patients (4, 5).The prevalence, characteristics and clinical outcomes of children in these units is largely unknown.

The paucity of data on the prevalence of pediatric critical care illness burden in LMICs, including Kenya, is a persistent problem (1, 4, 6, 7). With the lack of these data, estimates can be only inferred from data on reported pediatric mortality rates. Unfortunately, the country's public health care policy primarily focuses on collection of mortality data in children under 5 years of age**.** By 2022 Kenya's neonatal, infant and under–five mortality was 43, 21 and 41 per 1,000 live births (8). A 2013 study in Kenya revealed that 3.9% of in–hospital deaths were among children aged 5–17 years (9). The majority of these deaths stemmed from preventable and treatable causes such as pneumonia, diarrhoea, malaria, severe acute malnutrition, pre-term birth complications, birth asphyxia and trauma. Beyond preventive measures, offering emergency and critical care interventions, including an escalation of care where feasible, holds a strong potential to decrease mortality rates among children (3).

In Kenya, there are an estimated 196 pediatric and 254 neonatal intensive care unit (ICU) beds (8). Notably, Kenya's largest public hospital, Kenyatta National Hospital (KNH) in Nairobi, established the country's first public 5-bed pediatric intensive care unit (PICU) in 2015 (4). However, the available data lack specificity regarding the distribution, characteristics, and operational status of these beds. A recent study reported that approximately 24% of ICU beds in the country are non-functional with challenges in availability of trained personnel, infrastructure and equipment (10). The majority of these ICUs are run by anesthesiologists who are not formally trained in intensive care medicine. Equipment availability varied between facilities. Essential monitoring and hemodynamic support devices, especially invasive blood pressure monitors, capnography, and cardiac output measurement tools are rarely available across the country. Additionally, while most units reportedly have one ventilator per bed, approximately 70% of these ventilators are not equipped with pediatric-specific ventilation modes (10).

Studies conducted in high–income countries have consistently demonstrated the superiority of admitting children to dedicated PICUs over units intended for adult patients (11, 12). However, in the LMICs where these specialized units are scarce and available units limited in resources, contextually sensitive, prospective registry-based studies of pediatric admissions in the adult ICUs and HDUs could provide insight in the development and improvement of pediatric critical care initiatives with the available resources (13, 14). We, therefore, undertook this study to characterize the clinical features and outcomes of pediatric patients admitted to adult ICUs and HDUs within the Kenya Critical Care Registry.

## Methods

### Study design

The study was a prospective observational cohort study using data from the Kenya Critical Care Registry.

### Study setting

The Kenya Critical Care Registry, housed under the “Critical Care Society of Kenya” (CCSK), was started in 2020. It involves voluntary membership from any hospital in Kenya with either an ICU, a HDU or both. Currently the registry has both public and private hospitals from five different counties, and actively collects data from six intensive care units (ICU) and five high dependency units(HDU) in Kenya. None of these units are classified as pediatric intensive care units (PICUs) or neonatal intensive care units (NICUs). Combined, these units have a total of 94 beds. The characteristics of the eleven units are detailed in Supplementary Table S3.

### Ethical considerations

Ethics approval for the registry was sought from the Aga Khan University Institutional Ethics Review Committee, Mombasa, Kenya (2019/IERC-89, 26 November 2020). Ethical approval for the current analysis was obtained as part of the “Baseline Kenya Critical Care Registry output” (2021/IERC-125, 28 September, 2021) and the “National Commission for Science, Technology and Innovation” (NACOSTI) (16058). NACOSTI–accredited ethical committees provided further approval and waived the need for individual patient consent. The study is registered at clinicaltrials.gov (study identifier NCT05456217). This analysis results are reported following the Strengthening of the reporting of Observational Studies in Epidemiology (STROBE) statement checklist (Supplementary Table S1).

### Patient selection

We included all consecutive encounters of patients younger than 18 years who were admitted to ICUs or HDUs participating in the Kenya Critical Care Registry between January 2021 to June 2022.

### Data collection

A cloud–based platform was used to collect data. Quality assurance, data flow and data safety features of the registry platform used by Kenya Critical Care Registry are detailed in Supplementary Appendix 2. Dedicated data collectors entered data on the registry platform at different timepoints, namely patient ICU or HDU admission day, during the first 24 h of care, once daily during patient stay and at discharge from ICU or HDU. Intra and inter–user variability was minimized by remote training of data collectors and troubleshooting. All data collectors had protected time to perform the data collection and underwent regular remote training and troubleshooting facilitation through bi-weekly meetings. Individual site coordinators ensured the integrity and completeness of data collection. Source data verification was not performed on any registry site.

Admission diagnosis and surgical procedures were coded using the Systematized Nomenclature of Medicine Clinical Terms (SNOMED CT). Neurological status was assessed through the “Alert, Verbal, Pain and Unresponsive” (AVPU) scale, but only in patients that were not receiving sedative medication. On the AVPU scale, patients are classified as alert, responsive only to verbal stimuli, responsive only to painful stimuli, or unresponsive, with lower levels indicating more severe impairment of consciousness. For vital signs such as respiratory rate, heart rate and blood pressure the first reported value upon admission to the ICU was entered in the registry platform. For core laboratory parameters such as hemoglobin and platelet count, data collectors recorded the values measured within the first hour of admission to ICU. If unavailable, the last reported platelet count prior to admission was entered (maximum 24 h).

Data capturing included demographic data, including data to calculate the “Emergency Department–Pediatric Early Warning Score” (ED–PEWS) (15), main reasons for and source of admission, patient characteristics on and during admission in the unit, management features including organ support measures, follow–up data such as unit and hospital discharge, unit mortality, and duration of ventilation. The ED-PEWS was chosen due to the absence of adequate data to compute other pediatric severity scoring systems such as the Pediatric Risk of Mortality (PRISM), Pediatric Logistic Organ Dysfunction (PELOD) or the Pediatric Index of Mortality (PIM).

#### Outcomes

The primary outcome was mortality in the ICU or HDU. Secondary outcomes included clinical management features- organ support, use of sedative drugs, vital signs and laboratory measurement and the use of antimicrobials-, duration of mechanical ventilation, length of stay in ICU or HDU and risk factors for mortality.

#### Sample size calculation

We did not perform a formal sample size calculation. Given the exploratory nature of this registry-based observational study and the relative infrequency of pediatric ICU admissions, all eligible patients within the study period were included to maximize statistical power and generalizability.

#### Statistical analysis

Continuous variables are presented as medians with the interquartile range (IQRs), while categorical variables are presented as frequencies and proportions. Continuous data were tested for normality using the Shapiro–Wilk normality tests. Comparisons between groups were performed using a t–test for normally distributed continuous variables, the Wilcoxon–Mann–Whitney test for ordinal and non–normally distributed continuous variables, and the Fisher exact test for categorical variables. For missing data in categorical variables, we reported denominators and adjusted proportions accordingly.

Patients were categorized by medical or surgical admission. The primary endpoint, ICU or HDU mortality, was reported and compared between these two categories, and also presented for the age groups. The age group stratification was data-driven and classified as “neonates” (0–28 days), “infants” (<1 year), “toddlers” (1–5 years), “children” (5–12 years), and “teens” (> 12 years). The calculation of ED–PEWS was modified to account for the absence of capillary refill time and increased work of breathing (Supplementary Table S1).

Univariate analyses were performed to explore associations with mortality. Multivariable logistic regression models were then fitted to identify independent risk factors for ICU/HDU mortality, adjusting for potential confounders including age, sex, admission type, ventilation status, laboratory and vital parameters on admission. Covariates with *P* < 0.2 in univariable analysis and without evidence of collinearity were considered for inclusion. The participating center was treated as a random effect to account for variability across sites. A stepwise backward elimination approach was employed to refine the model, using a significance level of 0.05 as the threshold for variable retention. Missing data were handled using multiple imputation by chained equations (MICE). A total of *m* = 20 imputed datasets were generated, and analyses were performed separately on each dataset with estimates combined using Rubin's rules. Outcome variables were not imputed. Statistical analyses were performed using R (R Foundation for Statistical Computing, Vienna, Austria) with a *p* < 0.05 considered statistically significant.

## Results

### Patients

**Figure 1:**
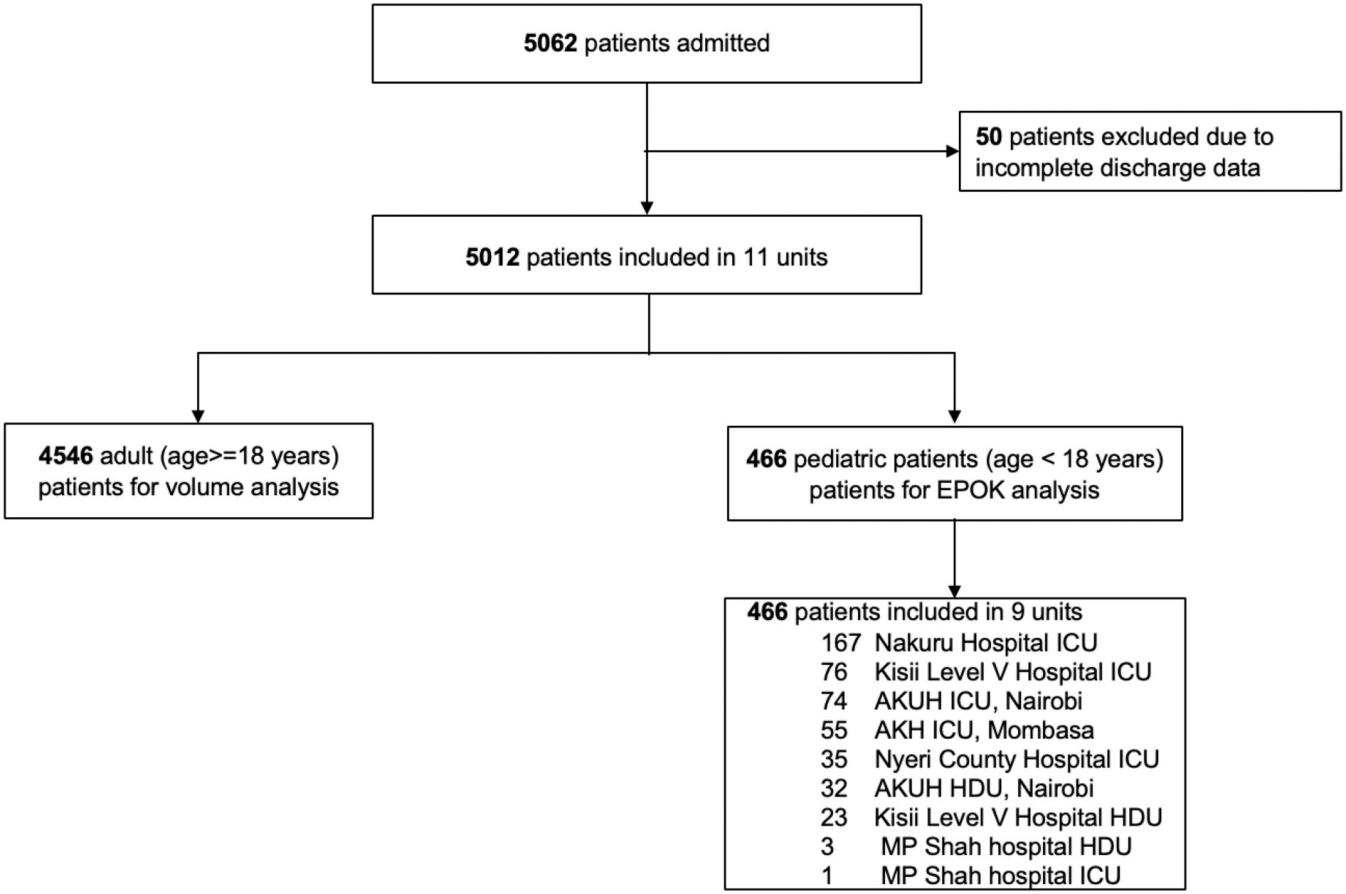
Patient flowchart. ICU, intensive care unit; HDU, high dependency unit; SARI, severe acute respiratory infection; AKUH, Aga Khan University Hospital.

Of the 5,012 ICU or HDU admissions, 466 (9.3%) involved critically ill children (Figure 1). The proportion of critically ill children remained stable over time (Supplemenatry Figure S1). The median age was 2.0 (0.4–9.0) years, with 173 children (37.1%) aged under one year (Table 1). Most cases were medical (76.6%), and included pneumonia, head injury, organophosphate poisoning and respiratory distress of the newborn as main admission codes (Supplementary Table S4**)**. Surgical admissions included emergency surgeries that were more prevalent as compared to planned surgeries. Explorative laparotomy and craniotomy were the most frequent surgical procedures (Supplementary Table S5). Median modified ED–PEWS was higher in medical patients compared to surgical patients (20 vs. 13; *p* = 0.01). A significantly higher proportion of medical patients were classified as unresponsive on the AVPU scale at admission compared to the surgical cohort (20.2% vs. 8.3%; *p* = <0.001).

**Table 1:** Demographic characteristics, admission sources, type of admission, severity of illness on admission, comorbidities and top 5 primary diagnoses of children admitted to adult ICUs.

Variables	All patients (*n* = 466)	Medical (*n* = 357)	Surgical (*n* = 109)	*P* value
Demographics				
Age in years	2.0 (0.4,9.0)	2.0 (0.3,9.0)	1.6 (0.5,6.0)	0.70
Age groups				0.01
Neonates	78 (16.7)	67 (18.8)	11 (10.1)	
Infants	95 (20.4)	65 (18.2)	30 (27.5)	
Toddlers	131 (28.1)	93 (26.1)	38 (34.9)	
Children	70 (15.0)	57 (16.0)	13 (11.9)	
Teens	92 (19.6)	75 (21.0)	17 (15.6)	
Sex, female	187 (40.1)	149 (41.7)	38 (34.9)	0.24
Admission source				<0.001
ED	97 (20.8)	94 (26.3)	3 (2.8)	
Ward	220 (47.2)	204 (57.1)	16 (14.7)	
Operating theatre	84 (18.0)	8 (2.2)	76 (69.7)	
ICU/HDU	23 (4.9)	14 (3.9)	9 (8.3)	
Other hospital	40 (8.6)	35 (9.8)	5 (4.6)	
Not recorded	2 (0.4)	2 (0.6)	0	
Type of admission				
Readmissions	15 (3.2)	12 (3.4)	3 (2.8)	0.99
Emergency surgery	63 (13.5)	N/A	63 (57.8)	–
Planned surgery	46 (9.9)	N/A	46 (42.2)	–
Severity on admission				
Modified ED-PEWS^a^	17 (9.0–24.0)	20 (11.0–25.0)	13 (6.0–20.5)	0.01
Neurological status				<0.001
Alert	181 (38.8)	131 (36.7)	50 (45.9)	
Verbal	14 (3.0)	8 (2.2)	6 (5.5)	
Pain	88 (18.9)	71 (19.9)	17 (15.6)	
Unresponsive	81 (17.4)	72 (20.2)	9 (8.3)	
Not assessed^b^	102 (21.9)	75 (21.0)	27 (24.8)	
Comorbidities				<0.001
Type 1 Diabetes	12 (2.6)	12 (3.4)	0	
Asthma	8 (1.7)	7 (2.0)	1 (0.9)	
Sickle cell	6 (1.3)	5 (1.4)	1 (0.9)	
Epilepsy	6 (1.3)	6 (1.7)	0	
Other neurological condition	3 (0.6)	3 (0.8)	0	
Tuberculosis	3 (0.6)	3 (0.8)	0	
Cardiovascular diseases	3 (0.6)	1 (0.3)	1 (0.9)	
AIDS	2 (0.4)	2 (0.6)	0	
Depression	2 (0.4)	1 (0.3)	1 (0.9)	
Leukemia	2 (0.4)	1 (0.3)	1 (0.9)	
Top 5 primary diagnoses	*N* (%)			
All patients, *n* = 466				
COVID Pneumonia	24 (5.2)			
Exploratory laparotomy	21 (4.5)			
Organophosphate poisoning	17 (3.6)			
Pneumonia	14 (3)			
Severe birth asphyxia	14 (3)			
Medical admission, *n* = 357				
COVID Pneumonia	24 (6.7)			
Organophosphate poisoning	16 (4.5)			
Severe birth asphyxia	14 (3.9)			
Neonatal sepsis	13 (3.6)			
Pneumonia	13 (3.6)			
Surgical, *n* = 109				
Exploratory laparotomy	21 (19.3)			
Craniotomy	5 (4.6)			
Exploration of laparotomy site	5 (4.6)			
Debridement	3 (2.8)			
Insertion of chest tube	3 (2.8)			

Data reported as median (interquartile range) or number (%). IQR, interquartile range; ICU, intensive care unit; HDU, high dependency unit; ED, emergency department; PEWS, pediatric early warning score.

a Modified ED-PEWS was available for 219 patients. It ranges from 0 to 53, was computed only for patients aged <16 years and modified to account for missing data as detailed in Supplementary Table S1.

b Patient sedated or data not recorded.

### Management features during the first 24 h

Half of the children received invasive ventilation while non-invasive ventilation was rarely used (Table 2). A minority of patients received vasoactive drugs and renal replacement therapy was uncommon. Approximately one in five children were sedated during the first 24 h of admission. More than 80% of children received at least one antibiotic, with a higher antibiotic prescription rate in the surgical group. Ceftriaxone was the most frequently prescribed drug in both groups, followed by metronidazole.

**Table 2:** Management features in the first 24 h in terms of organ support, sedation practice, laboratory parameters and antimicrobial use.

Variables	All children (*n* = 466)	Medical (*n* = 357)	Surgical (*n* = 109)	*p*-value
Organ support				
Ventilation status				0.089
Invasive mechanical ventilation	228 (48.9)	181 (50.7)	47 (43.1)	
Non-invasive ventilation or HFNT	7 (1.5)	7 (2.0)	0	
Spontaneously breathing	227 (48.7)	165 (46.2)	62 (56.9)	
Not recorded	4 (0.9)	4 (1.1)	0	
Cardiovascular support	6 (1.3)	5 (1.4)	1 (0.9)	0.84
Renal replacement therapy	6 (1.3)	6 (1.7)	0	0.34
Sedation^a^				
Use of sedative drugs, *n* = 228	88 (38.6)	69 (38.1)	19 (40.4)	0.90
Laboratory				
Platelet count, 10^3 per microliter	277 (184,407)	265 (176,376)	334.0 (228,461)	<0.001
White blood cells, 10⁹/L	11.6 (7.4,16.2)	11.7 (7.4,16.3)	10.9 (7.2,15.4)	0.59
Hemoglobin, g/dL	11.5 (9.5,13.6)	11.4 (9.4,13.6)	11.8 (9.8,13.5)	0.67
Antimicrobial use				
Use of antibiotics				<0.001
No antibiotic	75 (16.1)	63 (17.6)	12 (11.0)	
One antibiotic	224 (48.1)	180 (50.4)	44 (40.4)	
More than one antibiotic	163 (35.0)	110 (30.8)	53 (48.6)	
Not recorded	4 (0.9)	4 (1.1)	0	
Antibiotic type				
Ceftriaxone	182 (39.1)	127 (35.6)	53 (48.6)	0.006
Metronidazole	92 (19.7)	43 (12.0)	49 (45.0)	0.23
Ceftazidime	54 (11.6)	47 (13.2)	7 (6.4)	0.13
Flucloxacillin	36 (7.7)	36 (10.1)	0	–
Meropenem	33 (7.1)	20 (5.6)	7 (6.4)	0.99

Data reported as median (interquartile range) or number (%). Cardiovascular support specifically refers to the use of vasoactive medications. ICU, intensive care unit; HDU, high dependency unit; HFNT, high flow nasal therapy; bpm, breaths per minute.

a For sedation, the denominator is restricted to patients who were mechanically ventilated.

### Outcomes

The overall mortality rate in ICU and HDU was 34.5%, higher in medical admissions than in surgical admissions (39.5 vs. 18.3%, *p* < 0.001) (Table 3). Among the survivors, the median length of stay and the duration of mechanical ventilation was one day longer in medical than in surgical admissions (*p* = 0.001).

**Table 3:** Patients outcomes.

Variables	All children (*n* = 466)	Medical (*n* = 357)	Surgical (*n* = 109)	*P*-value
Mortality at ICU discharge	161 (34.5)	141 (39.5)	20 (18.3)	<0.001
Neonates, *n* = 78	50 (64.1)	46 (68.7)	4 (36.4)	0.08
Infants, *n* = 95	40 (42.1)	34 (52.3)	6 (20.0)	0.006
Toddlers, *n* = 131	35 (26.7)	29 (31.2)	6 (15.8)	0.11
Children, *n* = 70	20 (28.6)	18 (31.6)	2 (15.4)	0.40
Teens, *n* = 92	16 (17.4)	14 (18.7)	2 (11.8)	0.74
Discharge destination				
Ward	254 (83.3)	178 (82.4)	76 (85.4)	
HDU	18 (5.9)	11 (5.1)	7 (7.9)	
Other hospital	14 (3.0)	9 (4.2)	0	
Home	9 (3.0)	8 (3.7)	1 (1.1)	
ICU	6 (2.0)	2 (0.9)	4 (4.5)	
Other	4 (1.3)	3 (1.4)	1 (1.1)	
Length of stay in ICU, days	3 (2–6)	3 (2–6)	3 (2–5)	0.16
In survivors	3 (2–7)	4 (3–7)	3 (2–5)	0.001
In non-survivors	3 (2–5)	3 (2–5)	3.5 (2–7.3)	0.20
Duration of invasive mechanical ventilation, days	1 (1–4)	2 (1–4)	1 (1–1)	<0.001
In survivors	1 (1–4)	1 (1–6)	1 (1–1)	<0.001
In non-survivors	2 (1–4)	2 (1–4)	2 (1–3)	0.7

Data is presented as median (interquartile range) or number (percentage).

For mortality by age group, percentages are calculated using the total number of patients in each subgroup (e.g., neonates, infants, etc.) as the denominator.

### Factors associated with ICU mortality

**Figure 2:**
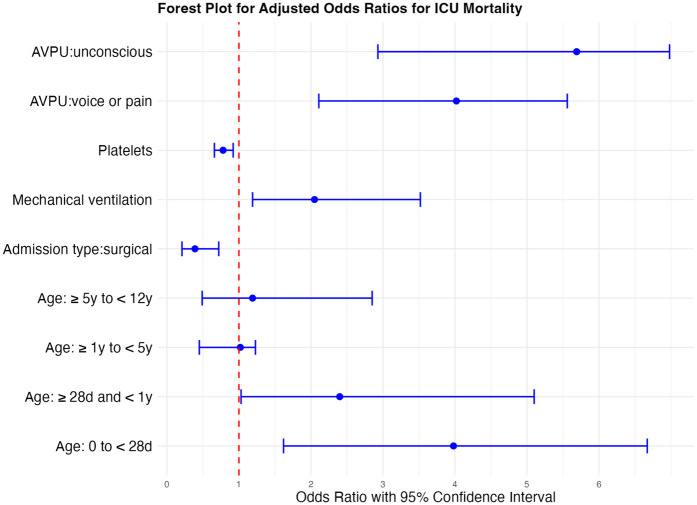
Forest plot of odds ratios for death in ICU for significant factors affecting ICU mortality. *Odds ratios (ORs) are presented with 95% confidence intervals. An OR < 1 (left of the reference line) indicates reduced odds of ICU mortality (favors survival), whereas an OR > 1 (right of the reference line) indicates increased odds of ICU mortality (favors death). OR reported for a change in 10.000 platelets.

Independent risk factors for mortality included age < 28 days (aOR 3.98, 95% CI 1.62–6.67; *p* = 0.002), admission due to a medical reason, unresponsiveness at admission (aOR, 5.69, 95% CI 2.93–6.98; *p* < 0.001), and receiving invasive ventilation (aOR 2.05, 95% CI 1.19–3.52; *p* < 0.001), while higher platelet count was associated with increased survival (aOR 0.78, 95% CI 0.66–0.92; *p* = 0.009) (Table 4 and Figure 2).

**Table 4:** Determinants of mortality in ICU.

Variables	Unadjusted analyses Odds ratio (95% CI)	*p*-value	Multivariable model Odds ratio (95%CI)	*p*-value
Case mix features				
Age group				
0 to <28 days (neonates)	8.48 (4.25,17.69)	<0.001*	**3.98 (1.62,6.67)**	** 0**.**002**
≥ 28 days to <1 year	3.45 (1.78,6.93)	<0.001*	**2.40 (1.03,5.10)**	**0**.**042**
≥ 1 year to <5 years	1.73 (0.90,3.43)	0.104	1.02 (0.45,1.23)	0.957
≥ 5 years to <12 years	1.90 (0.90,4.06)	0.092	1.19 (0.49,2.85)	0.827
≥ 12 years	Reference		Reference	
Sex			Not retained	
Female	Reference			
Male	0.61 (0.41,0.90)	0.014*		
Comorbidity				
None	Reference		Not retained	
At least one comorbidity	0.33 (0.16,0.62)	0.001*		
Admission type				
Medical	Reference		Reference	
Surgical	0.34 (0.19,0.57)	<0.001*	**0.39 (0.21,0.72)**	**0**.**002**
Management features (first 24 h)				
Use of MV	4.64 (3.08,7.11)	<0.001*	**2.05 (1.19, 3.52)**	**0**.**007**
Sedated on admission	1.30 (0.82,2.05)	0.254	–	
Use of antibiotics			Not retained	
None	Reference			
At least one antibiotic	2.74 (1.54,5.14)	0.001*		
LABORATORY				
PaO_2_/FiO_2_	0.99 (0.99,1.00)	0.155	Not retained	
Hemoglobin (g/dL)	1.02 (0.94,1.09)	0.589	–	
Platelets count (10^4/µL)*	0.72 (0.61,0.84)	<0.001*	**0.78 (0.66,0.92)**	**0**.**009**
White blood cells count (/mm^3^)	1.02 (1.00,1.04)	0.058	Not retained	
VITAL SIGNS				
Neurological status on admission				
Alert	Reference		Reference	
Voice or Pain	4.12 (2.40,7.15)	<0.001*	**4.02 (2.11,5.56)**	**<0**.**001**
Unconscious	7.48 (4.60,12.41)	<0.001*	**5.69 (2.93,6.98)**	**<0**.**001**
Respiratory rate	1.03 (1.01,1.04)	<0.001*	Not retained	
Heart rate (bpm)	1.00 (1.00,1.01)	<0.001*	Not retained	
MAP (mm Hg)	0.97 (0.96,0.98)	<0.001*	Not retained	

* OR reported for a change in 10.000 platelets. Variables with *p* < 0.20 in univariate analysis and without collinearity were included in the initial multivariable model. “Not retained” indicates variables excluded during backward elimination (*p* ≥ 0.05). Bold values indicate significant predictors of mortality in the multivariable analysis.

## Discussion

The findings of this analysis can be summarized as follows: (1) One in ten admissions to the units involves a child; (2) a large proportion of these children are younger than one year; (3) most are admitted with a medical diagnosis and tend to be more severely ill; (4) the mortality rates are high; (5) children admitted for medical reasons and those younger than one year face an increased risk of a poor outcome; and (6) all identified risk factors for a poor outcome are non–modifiable.

The lack of adequate ICU beds and specialized pediatric critical care units persists in resource-limited countries (10, 16). Though the consistent volume of pediatric admissions observed in this study indicates a system that requires centralization of severely ill pediatric patients (4), the establishment of pediatric ICUs may not be feasible in the short term (3). The interpretation of outcomes of interest including survival, events of interest, patient-reported outcomes and resource utilization of pediatric admissions in adult ICUs could help inform efforts in improvement of care and outcomes (14). This study provides a comprehensive picture of children being escalated to adult critical care units in Kenya, forming a crucial basis for informing care in these units.

The age distribution of pediatric admissions to ICUs and HDUs in our registry mirrors other studies, with a prevalence of infants (17–19). Pneumonia was the leading cause of admission, consistent with findings from pediatric ICUs in similar settings, though differing from reports in South Africa, Malawi and Ethiopia (4, 17, 20–23). The predominance of pneumonia as the primary reason for admission to ICUs and HDUs in LMIC (4, 5, 17, 18, 24) underscores the substantial burden of acute respiratory infection as a leading cause of morbidity and mortality in children across the region (2, 25, 26). It is commendable that there are government efforts to prevent pneumonia through vaccination. Strategies aimed at improving access to care and prompt treatment could mitigate the progression and subsequent need for respiratory support in childhood pneumonia (27). Pediatric focussed training of personnel in adult ICUs and the development of treatment protocols can improve outcomes for children admitted with pneumonia in adult ICUs (28).

The reported incidence of sepsis as an admission diagnosis was notably lower than in other global studies (22, 29–32). The study was conducted prior to the recent development and validation of the Phoenix Sepsis Criteria, at a time when the diagnosis of sepsis in LMICs was limited by the lack of applicability and specificity of the pediatric criteria for sepsis (33). An early identification of sepsis is crucial for best-practice sepsis including the unnecessary use of antimicrobials (34). In this study, there was a mismatch between the diagnosis of sepsis and the antibiotic prescription patterns. Microbiological culture data was unfortunately not available to aid in the interpretation of these patterns.

The mortality rate was comparable with that from an earlier Kenyan study and South African data on children ventilated outside ICU settings (5, 21). On the contrary, Western Cape, South Africa, reported a much lower mortality rate (17, 18), though children requiring more than 72 h of ventilation were transferred to a tertiary PICU. The overall mortality rate was similar to that of adults admitted during the same period (35) though the mortality rate was much higher in children less than one year with a medical diagnosis at admission. Notably, the mortality rate in our cohort appears higher than that reported by a PICU in Kenya and other parts of Africa (4, 22, 29, 30). It can be inferred therefore that in a setting with scarce PICUs, priority can be given to the infants with a medical condition over the surgical older children (19, 22, 30).

The high incidence of respiratory illness and altered level of consciousness upon admission could explain the extensive use of invasive ventilation within the first 24 h of care. In Kenya, the infrequent use of inotropic support and noninvasive ventilation (NIV) is not unique to the pediatric population (35). The underuse of NIV and high flow nasal oxygen (HFNO) contrasts with global trends in critical care (36). While NIV has proven beneficial in neonates, data on its use in the non–neonatal population in LMICs remains scarce (37, 38). This is an area of consideration in future studies.

There were independent associations between the primary outcome, ICU or HDU death, and AVPU score, receipt of invasive ventilation, platelet count, and age under 28 days. Among these, AVPU score appeared to have the strongest association. Though all the identified risk factors for mortality are nonmodifiable, these can be used to inform risk stratification, triage protocols and early warning systems.

### Strengths and limitations

The strength of our analysis lies in its registry–based structure and robust coverage of admissions, as every child entering the ICUs or HDUs was intercepted by the clinical quality database. Also, the registry data was prospectively collected in a near real–time manner using a validated common data model and cloud–based infrastructure. There was a representative balance between public and private units with a geographical representation that mirrors the nationwide distribution of critical care units more prevalent in large metropolitan areas and in the south of the country (9), enhancing the generalizability of the findings and ensuring that the results are applicable across different healthcare settings. Furthermore, the registry's design allows for a near–complete capture of data when these are obtained as part of routine care, minimizing the risk of selection bias. Follow–up was also near complete, further strengthening the reliability of the outcomes reported.

This analysis has several limitations. Firstly, it is restricted to children admitted to adult ICUs and HDUs, preventing comparisons of epidemiology, patient characteristics, and outcomes with those of children cared for in dedicated pediatric units. Secondly, the absence of pediatric–specific severity scores limits the ability to describe illness severity at admission and to compute risk–adjusted outcomes. Thirdly, data collection was limited to information recorded for clinical purposes meaning that key variables such as ventilator settings, vasopressor doses, and other treatment details that may contribute to outcome prediction were not available. Additionally, mortality was limited to the unit with no post discharge outcomes. We lacked data on the specific causes of death, which may differ from those seen in high-income settings and could influence interpretation of mortality patterns. Finally, as this is an observational study, unmeasured confounders may have influenced the findings, and causal inferences cannot be drawn.

## Conclusion

In a representative sample of adult Kenyan ICUs and HDUs, 9.3% of admissions were children who often receive invasive ventilation and have a high crude mortality rate. This analysis highlighted several scarcely modifiable factors that can orient early triage and escalation of care. Our findings highlight the need for targeted investments in pediatric-specific critical care infrastructure including early recognition protocols, trained personnel within the adult HDUs and ICUs in LMICs. Further research should focus on refining the identification of potentially modifiable risk factors for mortality that can drive changes in clinical management and quality improvement initiatives.

## Data Availability

The original contributions presented in the study are included in the article/Supplementary Material, further inquiries can be directed to the corresponding author.
