# Supplementary material for: Clinical characteristics and outcomes of children admitted to adult intensive care and high-dependency units in Kenya: a multicenter registry-based analysis
Source: Front Pediatr. 2025 Dec 5;13:1672012. doi: 10.3389/fped.2025.1672012 (PMC12716317; doi:10.3389/fped.2025.1672012)

# SUPPLEMENTARY MATERIAL TO:

**Clinical Characteristics and Outcomes of Children Admitted to Adult Intensive care and High-Dependency Units in Kenya: A Multicenter Registry-Based Analysis**

## Contents


Quality assurance and data safety 2

eFigure 1. Volume of pediatric ICU admissions recorded in the Kenya Critical Care Registry 3

eTable 1. Modified ED PEWS computation 4

eTable 2. Registry dataset variables and amount of missing data 5

eTable 3: Characteristics of participating units 6

eTable 4. Detail of medical disorders coded in the EPOK-Ped cohort 7

eTable 5. Detail of surgical procedures coded in the EPOK-Ped cohort 8

eTable 6. Determinants of death in ICU 9

**Quality assurance and data safety**

All registry data is housed and stored securely on a national server located on Kenyan soil. Once entered electronically on password-protected computers at participating facilities, it is automatically encrypted before it leaves the institution for the visualization loop within a ring-fenced server at NICS, where it is unencrypted and aggregated for automated visualization. NICS follows healthcare standard GDP and HIPAA standards. The processed data is then re-encrypted before it is transferred back to the national server, where it is automatically unencrypted and available for review by authorized personnel, through a secure two step log in - a process that is navigated with the help of Kenyan IT teams. An audit trail is created any time the registry is accessed, to see who has logged into it, when, and what data was retrieved or modified. The data processing stage is fully automated and patient data is therefore not viewable by anyone outside of participating facilities. The team of investigators are only able to view de-identified aggregate data whose source institution is not identifiable. The NICS team have access to the system only for purposes of system support and maintenance and are not able to visualise any patient data.

Access to the electronic registry was restricted to personnel authorized by the leadership at participating facilities, each of whom have been provided with a unique login and password for this purpose. Each person with such access to the registry signed a data protection agreement, indicating that they will not share their login details with anyone else, and that they will not share the contents of the registry with unauthorized personnel. As in other ICU registries, identifiable patient data was restricted to hospital staff authorized to access the critical care registry by the administration of individual facilities. The data extraction for analysis only concerned de-identified data.

**eFigure 1. Volume of pediatric ICU admissions recorded in the Kenya Critical Care Registry**

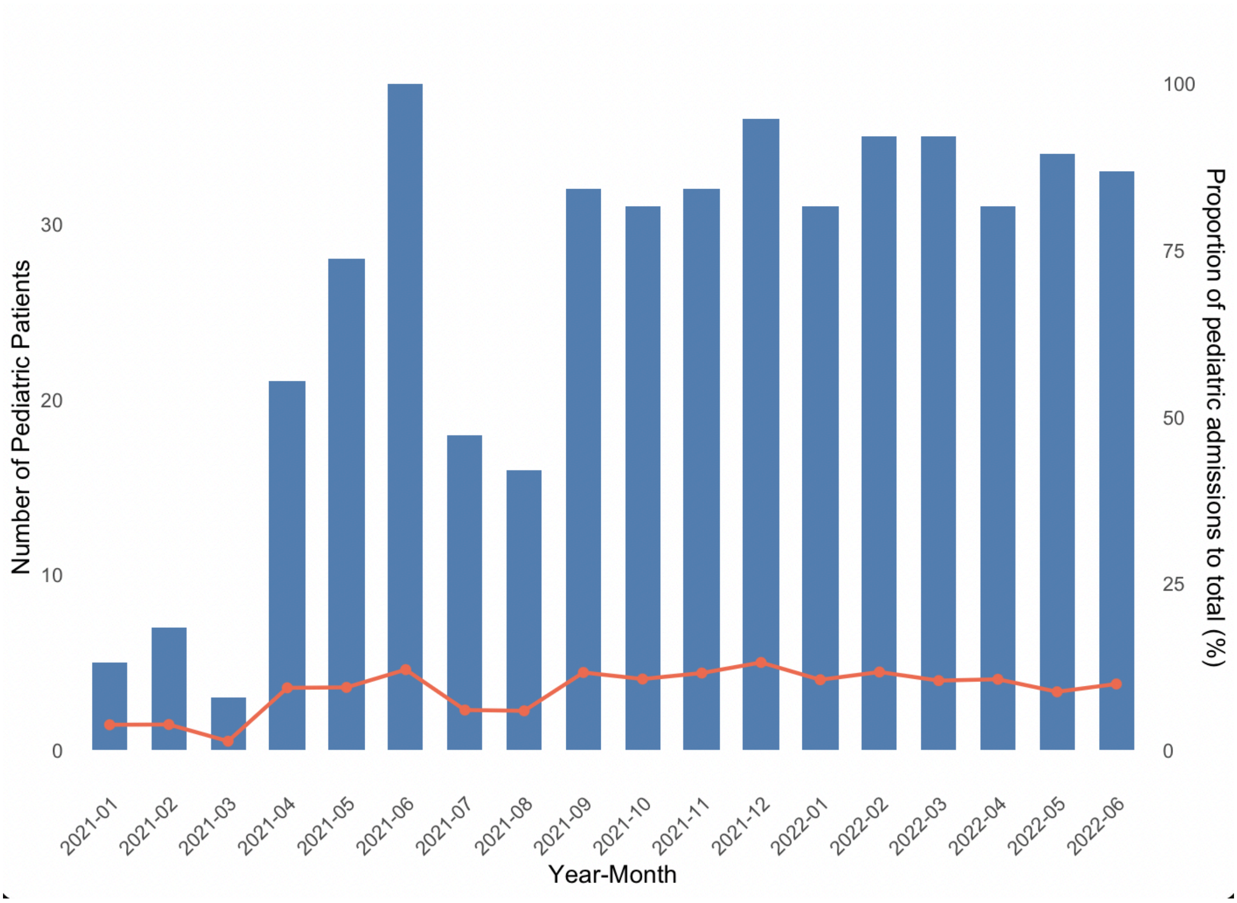


-

## Registry dataset

### Core variables

These variables provide essential information to enable reporting of case mix, acuity, and risk adjusted outcome. The patient identifiers are used to enable individual unit stakeholders to track patients during the encounter. These are removed during data curation and an encounter ID is generated. All core variables are mandatory by default.

### On admission

Completed as close as possible to the time of admission of the patient to the unit. Information is as recorded at admission.

| Variable name | Guidance for collection of variables |
|---|---|
| Section: Patient information | |
| Patient's name* | The patient's surname and their given name. |
| Medical record number* | A number given to a patient for a specific hospital encounter or repeated encounters. |
| Age* | Age of the patient on the day of admission to the unit. |
| Sex* | The patient's gender as identified at birth. |
| National identity number | A unique identifier for a patient that is given by the government. |
| Contact number | The phone number of the patient that can be used for contact. |
| Fee paying | Indicate whether the patient is paying fees. |
| Fee paying method | Type of fee paying method. |
| Other fee paying method | Specify the other type of fee paying method. |
| Comorbidities* | Select conditions currently present and diagnosed prior to this hospital admission. The list of options is mapped to the Charlson Comorbidity Index. Other comorbidities that may be important to the clinical team and can be input via the field 'Comorbidities (Other)'. |

| | |
|---|---|
| Other comorbidity* | Search the SNOMED CT1 list and select any other conditions that are not available in the comorbidities dropdown list. |

| Section: Admission details | |
|---|---|
| Date of hospital admission* | The date of arrival of the patient in the hospital. |
| Time of hospital admission* | The time of arrival of the patient in the hospital. |
| Date of unit admission* | The date of arrival of the patient in the unit. |
| Time of unit admission* | The time of arrival of the patient in the unit. |
| Unit admission source* | The patient's clinical location immediately prior to this unit admission. |
| Mode of arrival | The mode of transportation the patient used to arrive at the unit. |
| Readmission* | This unit admission encounter is a readmission: defined as; patient was discharged and readmitted to the Unit within the same hospital care episode. |
| Date of previous discharge | Previous unit discharge date during the patient's current hospital admission. |
| Type of admission* | Non operative: Patients not arriving directly from the operating theatre or recovery. This includes patients who may have undergone an operative procedure earlier in their hospital encounter.<br><br>Post operative: Patients who have been admitted to the unit directly from the operation theatre or recovery unit. |

| | |
|---|---|
| Emergency surgery* | Emergency surgery is defined as immediate surgery, where resuscitation (stabilisation and physiological optimisation) is simultaneous with surgical treatment and where surgery normally takes place within minutes of decision to operate. |
| Reason for admission (Operation)* | Search for the operative procedure from the SNOMED CT1 list which most accurately describes what procedure was undertaken that prompted the Unit admission. If there have been multiple operative |

| | |
|---|---|
| Reason for admission (Disorder)* | In addition to the operative procedure, search and select any disorders from the SNOMED CT list which necessitated admission to the Unit alongside the operation. |
| Reason for admission (Disorder) | The primary disorder (disease, condition, injury) which has necessitated admission to the unit. This should be based only on what is known or suspected to be the primary reason for admission. Any additional disorders (disease, conditions or injuries) deemed to be significant to the patient's admission to the unit, identified or presenting following the unit admission, should be added using the additional reason for admission fields, at any time during the unit encounter. The chronology of the disorders entered is retained in the data export. The primary disorder known at admission is used to determine severity of illness and prediction of mortality. |
| Severe acute respiratory infection* | Does the patient have community acquired pneumonia? This variable is used to link with public health surveillance, and disease specific studies. |

Assessment (24 hr post admission)

Completed 24 hours after Unit admission. This form reflects events, treatments and assessments occurring in the 24 hour period following unit admission.

| Variable name | Guidance for collection of variables |
|---|---|
| Section: Respiratory | |

| | |
|---|---|
| Ventilation* | Self vent: No breaths are delivered by a mechanical device during the first 24 hours after Unit admission (this includes breathing by own airway [+/- supplemental oxygen], breathing independently via tracheostomy, and CPAP, HFNC).<br><br>Mechanical vent: All or some of the breaths or a portion of the breaths are delivered by a mechanical device during the first 24 hours after Unit admission. |

| | |
|---|---|
| Route of ventilation* | The type of mechanically assisted breathing during the first 24 hours after Unit admission. If multiple modes are used, please report the most invasive (e.g, NIV being the least invasive, and tracheostomy being the most invasive). |
| Route of ventilation* | The primary route used by the patient for self-initiated breathing during the first 24 hours after Unit admission. If multiple modes are used, please report the highest support. |
| Oxygen delivery device* | The oxygen delivery device used by the patient during the first 24 hours after Unit admission. If multiple modes are used, please report the highest support. |
| $PaO_2$ | The partial pressure of oxygen measured in the arterial blood. The worst recorded value during the first 24 hours after Unit admission. |
| $SpO_2$ | Percentage of oxygen-saturated haemoglobin corresponding to the worst $PaO_2$ recorded. If $PaO_2$ is not recorded, then the worst recorded value during the first 24 hours after Unit admission. |
| $FiO_2$ | The inspired oxygen concentration delivered at the time of the worst $PaO_2$ recorded. If $PaO_2$ is not recorded, then enter the inspired oxygen concentration recorded at the time of the worst $SpO_2$. |
| $PaCO_2$ | The partial pressure of carbon dioxide measured in the arterial blood. The worst recorded value during the first 24 hours after Unit admission. |
| Arterial pH | The measurement of the pH of plasma of an arterial blood sample. The most deranged reading recorded during the first 24 hours after Unit admission. This is the reading furthest from the normal range of pH 7.35 to 7.45. |

| | |
|---|---|
| Respiratory rate* | Worst recorded respiratory rate during the first 24 hours after Unit admission. |
| Section: Cardiovascular System | |
| Heart rate* | Worst recorded heart rate during the first 24 hours after Unit admission. |
| Systolic blood pressure* | Worst recorded systolic blood pressure during the first 24 hours after |
| | Unit admission |
| Diastolic blood pressure* | Worst recorded diastolic blood pressure during the first 24 hours after Unit admission |
| Temperature* | Worst recorded temperature during the first 24 hours after Unit admission. |
| Cardiovascular support* | The use of continuous intravenous vasoactive medication during the first 24 hours after Unit admission. |
| Vasoactive therapy* | The type and dose of vasoactive drug used from the list of options. |
| Section: Neurological | |
| Sedated* | Use of sedative drugs for a minimum of one hour continuous infusion, or greater than one bolus. |
| Glasgow coma scale (eye)* | The worst (lowest) recorded GCS during the first 24 hours after Unit admission. If the patient is sedated, and or mechanically ventilated then it is the score immediately prior to sedation/ ventilation. |
| Glasgow coma scale (verbal)* | The worst (lowest) recorded GCS during the first 24 hours after Unit admission. If the patient is sedated, and or mechanically ventilated then it is the score immediately prior to sedation/ ventilation. |
| Glasgow coma scale (motor)* | The worst (lowest) recorded GCS during the first 24 hours after Unit admission. If the patient is sedated, and or mechanically ventilated then it is the score immediately prior to sedation/ ventilation. |

| | |
|---|---|
| AVPU Scale | The worst recorded AVPU during the first 24 hours after Unit admission. If the patient is sedated, and or mechanically ventilated then it is the score immediately prior to sedation/ ventilation.<br>[*We recommend using GCS as the primary variable to measure conscious level as this score enables derivation of severity of illness. If GCS is not measured as standard practice in the Unit, AVPU scale* |

| | |
|---|---|
| | *may be used as an alternative. If selected, mandatory status should be adjusted accordingly.*]<br>Messages addressed to "meeting group chat" will also appear in the meeting group chat in Team Chat |
| Section: Renal / Genitourinary | |
| Renal replacement therapy* | Use of renal replacement therapy during the first 24 hours after Unit admission. Any duration of RRT is considered as a 'Yes'. |
| Section: Laboratory Results | |
| Haemoglobin | The lowest recorded haemoglobin measured during the first 24 hours after Unit admission. If unavailable, provide the haemoglobin reported prior to admission (max 24 hrs prior). |
| Platelet count | The lowest recorded platelet count during the first 24 hours after Unit admission. If unavailable, provide the platelet count reported prior to admission (max 24 hrs prior). |
| Packed cell volume | Also called the haematocrit. This is the volume percentage of red blood cells (RBC) in blood. Please record the lowest value during the first 24 hours after Unit admission. If unavailable, provide the PCV reported prior to admission (max 24 hrs prior). |
| Serum sodium | The worst blood sodium recorded during the first 24 hours after Unit admission. If unavailable, provide the blood sodium reported prior to admission (max 24 hrs prior). |
| Serum potassium | The worst blood potassium during the first 24 hours after Unit admission. If unavailable, provide the blood potassium reported prior to admission (max 24 hrs prior). |

| | |
|---|---|
| Serum HCO3 | The worst blood bicarbonate recorded during the first 24 hours after Unit admission. If unavailable, provide the blood bicarbonate reported prior to admission (max 24 hrs prior). |
| Serum creatinine | The worst blood creatinine measured during the first 24 hours after Unit admission. If unavailable, provide the last reported blood creatinine prior to admission (max 24 hrs prior). |
| Serum bilirubin | The worst blood bilirubin measured during the first 24 hours after Unit |

**Unit outcome**

Completed following Unit discharge.

| Variable name | Data entry format | Dependency | Guidance for collection of variables |
|---|---|---|---|
| Date of Unit discharge* | Date [dd/mm/yyyy] | | The date on which the patient is discharged from or dies in the Unit. |
| Time of Unit discharge* | Time [hh:mm] | | The time at which the patient is discharged from or dies in the Unit. |
| Section: Clinical outcomes | | | |
| Unit discharge status* | Dropdown ['Alive', 'Dead'] | | Whether the patient is alive at the point of Unit discharge. |
| Unit discharge destination* | Dropdown ['Home', 'Other ICU', 'HDU', 'Ward', 'Transfer for specialist care', 'Discharge home for end of life care', 'Other hospital', 'Other', 'Nursing home or long-term care facility', 'Rehabilitation hospital'] | Unit discharge status = ‘Alive’ | Indication of the location the patient is destined for immediately after Unit discharge. |
| Decision to discharge* | Dropdown [‘Left against medical advice’, 'Patient request', 'Clinician decision'] | Unit discharge status = ‘Alive’ | Specify who made the decision to discharge the patient. |
| Treatment limitations during Unit stay* | Dropdown ['Withdrawal of treatment', 'Limitation of treatment', 'No limitation'] | Unit discharge status = ‘Alive’ | Indicate whether there was withdrawal of or limitations on treatment. |
| Type of treatment limitation during Unit stay* | Dropdown ['Not for intubation', 'Not for renal replacement therapy', 'Not for Unit readmission', 'Not for vasopressors'] | Treatment limitations = ‘’Limitation of treatment’’ | Select the type of treatment limitation. |

| | | | |
|---|---|---|---|
| Section: Intervention | | | |
| CPR performed * | Yes/No | | Score if the patient has had CPR (heart massage) during the Unit episode of care. Cardioversion without heart massage does not apply as CPR. |
| Section: Other | | | |
| Discharge note | String | | Any notes about the patient at the time of discharge or any post-discharge care instructions. |

Hospital outcome

Completed following hospital discharge.

| Variable name | Data entry format | Dependency | Guidance for collection of variables |
|---|---|---|---|
| Date of hospital discharge* | Date [dd/mm/yyyy] | | The date on which the patient is discharged from-, or dies in the hospital. |
| Time of hospital discharge* | Time [hh:mm] | | The time at which the patient is discharged from-, or dies in the hospital. |
| Section: Clinical outcomes | | | |
| Hospital discharge status* | Dropdown ['Alive', 'Dead'] | | Whether the patient is alive at the point of hospital discharge |
| Hospital discharge destination* | Dropdown ['Home', 'Other ICU', 'Transfer for specialist care', 'Discharge home for end of life care', 'Nursing home or long-term care facility', 'Rehabilitation hospital', 'Transfer to another acute hospital', 'Other'] | Hospital discharge status = ‘Alive’ | Indication of the location the patient is destined for immediately after hospital discharge. |

**eTable 1. Modified ED PEWS computation**
This score was modified from Zachariasse et al. due to the non-availability of two variables, ‘increased work of breathing’ and ‘increased capillary refill time’. Hence the range for the used score is **0 to 53.**

| **Variable** | | **Points** |
|---|---|---|
| Patient age (years) | 0-4 | 0 |
| | 5-11 | 4 |
| | 12-16 | 6 |
| Decreased consciousness* | No | 0 |
| | Yes | 14 |
| Respiratory rate (breaths per minute) | <30 | 0 |
| | 30-39 | 3 |
| | 40-59 | 5 |
| | ≥ 60 | 9 |
| Oxygen saturation (%) | ≥ 98 | 0 |
| | 94-97 | 4 |
| | 88-93 | 9 |
| | <88 | 15 |
| Heart rate (beats per minute) | <100 | 0 |
| | 100-139 | 3 |
| | 140-179 | 6 |
| | ≥ 180 | 9 |

*In this analysis defined as AVPU different from A (alert).

**Zachariasse JM, Nieboer D, Maconochie IK, *et al.*** Development and validation of a Paediatric Early Warning Score for use in the emergency department: a multicentre study. *Lancet Child Adolesc Health* 2020; 4: 583–91.

**eTable 2. Registry dataset variables and amount of missing data**
In **bold** variables used in the multivariate modelling analysis.

| Field name | Missing N (%) N=466 | Field name | Missing data N(%) |
|---|---|---|---|
| **Age** | **0 (0%)** | Blood glucose* | 165 (35.4%) |
| **Sex** | **0 (0%)** | Pain score* | 14 (3.0%) |
| Date of hospital admission | 0 (0%) | **Haemoglobin** | **108 (23.2%)** |
| Time of hospital admission* | 0 (0%) | **Platelets** | **109 (23.4%)** |
| Date of ICU admission | 0 (0%) | Packed cell volume | 157 (33.7%) |
| Time of ICU admission* | 0 (0%) | **White blood cell count** | **109 (23.4%)** |
| ICU admission source | 0 (0%) | Serum Sodium | 160 (34.3%) |
| Readmission | 0 (0%) | Serum Potassium | 170 (36.4%) |
| Date of previous discharge* | 5 (1.1%) | Serum $HCO_3$ | 341 (73.2%) |
| **Diagnosis type** | **0 (0%)** | Serum Creatinine | 220 (47.2%) |
| Emergency Surgery | 0 (0%) | Serum bilirubin | 355 (76.2%) |
| Reason for admission (operative or disorder) | 0 (0%) | Blood urea | 215 (46.1%) |
| **Comorbidities** | **0 (0%)** | **Oxygen saturation ($SpO_2$)** | **245 (52.6%)** |
| **Ventilation** | **4 (0.9%)** | | |
| Route of Mechanical ventilation | 4 (0.9%) | Date of discharge | 1 (0.2%) |
| High flow oxygen | 245 (53.8%) | Time of discharge | 1 (0.2%) |
| **$FiO_2$** | **59 (12.7%)** | Discharge status | 0 (0%) |
| **$PaO_2$** | **354 (76.0%)** | Discharge destination | 0 (0%) |
| Arterial pH | 354 (76.0%) | CPR status* | 1 (0.2%) |
| Sedated | 4 (0.9%) | | |
| Cardiovascular support | 4 (0.9%) | Left against medical advice | 0 (0%) |
| Renal replacement therapy | 4 (0.9%) | Discharge upon patient request | 0 (0%) |
| Antimicrobial use | 4 (0.9%) | Date of hospital discharge | 182 (39.1%) |
| **Systolic blood pressure** | **12 (2.5%)** | Hospital discharge status | 178 (38.2%) |
| **Diastolic blood pressure** | **12 (2.5%)** | | |
| **Respiratory rate** | **4 (0.9%)** | | |
| **Heart rate** | **4 (0.9%)** | | |
| Temperature | 4 (0.9%) | | |
| **AVPU condition** | **32 (6.9%)** | | |
| | | | |

*SpO2 is not collected on the admission assessment form but in the first daily form

## eTable 3: Characteristics of participating units

| Name of Hospital | AKH Mixed ICU, Mombasa | Mp Shah ICU, Nairobi | MP Shah HDU, Nairobi | AKUH Mixed ICU, Nairobi | AKUH HDU, Nairobi | Nakuru hospital Mixed ICU | Nyeri County Referral Hospital Mixed ICU | Kisii Teaching and Referral hospital ICU | Kisii Teaching and Referral hospital HDU |
|---|---|---|---|---|---|---|---|---|---|
| **ORGANIZATION** | | | | | | | | | |
| Hospital status | PNFP | PNFP | PNFP | PNFP | PNFP | Public | Public | Public | Public |
| Number of beds in Hospital | 143 | 250 | 250 | 258 | 258 | 1000 | 250 | 650 | 650 |
| Number of beds in unit | 4 | 7 | 9 | 11 | 16 | 9 | 6 | 6 | 3 |
| Yearly admissions | 123 | 180 | NA | 500 | 1500 | 300 | 165 | 122 | 27 |
| Model of care | Open | Open | Open | Open | Open | Open | Closed | Open | Open |
| **STAFFING** | | | | | | | | | |
| Specialty of in-charge | Ane | Ane | Ane | Med | Med | Ane | Ane | Ane | Ane |
| Availability of dedicated clinician | NC | C | C | NC | NC | No | No | NC | No |
| Number of nurses in the unit | 13 | 36 | 24 | 46 | 44 | 30 | 15 | 29 | |
| Nurse to bed ratio day time | 1:2 | 1:1 | 1:2 | 1:1 | 1:2 | 1:2 | 1:3 | 1:2 | 1:2 |
| Nutritionist | On consult | On consult | On consult | On consult | On consult | Dedicated | On consult | Dedicated | On consult |
| Physiotherapist | On consult | On consult | On consult | On consult | On consult | Dedicated | On consult | Dedicated | On consult |
| Counsellor or Psychologist | On consult | On consult | On consult | On consult | On consult | On consult | On consult | No | no |
| Pharmacist | On consult | On consult | On consult | Dedicated | On consult | On consult | On consult | On consult | On consult |
| **RESOURCES** | | | | | | | | | |
| Isolation rooms in the unit | none | 2 | 9 | 11 | 4 | 1 | 1 | 1 | 0 |
| Functional Mechanical Ventilators | 5 | 8 | 2 | 11 | 3 | 20 | 14 | 12 | 0 |
| Functional HFNT machines | 3 | 5 | 2 | 5 | 2 | 0 | 0 | 0 | 0 |
| Blood gas analysis machine | Yes | Yes | Yes | Yes | Yes | Yes | Yes | Yes | No |

NC, non consultant doctor; C, consultant; CCN, critical care nursing; HFNT, high flow nasal therapy; ICU, intensive care unit; HDU, high dependency unit.

**eTable 4. Detail of medical disorders coded in the EPOK-Ped cohort**

| Type of disorder | Number of patients | Type of disorder | Number of patients |
|---|---|---|---|
| Covid Pneumonia | 25 | Viral pneumonia | 2 |
| Pneumonia | 21 | Atypical pneumonia | 1 |
| Respiratory distress of newborn | 19 | Seizure disorder | 1 |
| Organophosphate poisoning | 19 | Croup | 1 |
| Diabetic ketoacidosis (DKA) | 18 | Otitis media | 1 |
| Unspecified head injury | 17 | Necrotizing enterocolitis | 1 |
| Severe birth asphyxia | 14 | Malaria | 1 |
| Neonatal sepsis | 13 | Hypoxic ischemic brain injury | 1 |
| Status epilepticus | 13 | Congenital brain edema | 1 |
| Aspiration pneumonia | 12 | Congenital stenosis of spinal canal | 1 |
| Severe (major) head injury | 12 | Myelomeningocele | 1 |
| Meningitis | 11 | Ependymoma | 1 |
| Sepsis | 9 | Tuberculous meningitis | 1 |
| Meconium aspiration | 7 | Tuberculoma | 1 |
| Burns | 7 | Transient ischemic attack | 1 |
| Febrile convulsions | 6 | Leigh’s disease | 1 |
| Mild birth asphyxia | 5 | Chronic migraine | 1 |
| Extremely low birth weight (ELBW) infant | 5 | Depression | 1 |
| *Missing* | 5 | Berry aneurysm | 1 |
| Acute respiratory distress syndrome (ARDS) | 4 | Hyperglycemia | 1 |
| Meningoencephalitis | 4 | Type 1 diabetes mellitus | 1 |
| Convulsive disorder | 4 | Myasthenia gravis | 1 |
| Acute respiratory failure | 3 | Acute drug intoxication | 1 |
| Retroperitoneal abscess | 3 | Alcohol intoxication | 1 |
| Psychogenic seizures | 3 | Fracture femur | 1 |
| Guillain-Barre syndrome (GBS) | 3 | Occipital bone fracture | 1 |
| Severe dehydration | 3 | Multiple trauma | 1 |
| Moderate birth asphyxia | 2 | Intestinal obstruction | 1 |
| Asthma | 2 | Post craniotomy | 1 |
| Acute respiratory infection (unspecified) | 2 | Immune thrombocytopenia | 1 |
| Pulmonary edema | 2 | Lymphoma | 1 |
| Septic shock | 2 | Leptomeningitis | 1 |
| Urinary tract infection | 2 | Kawasaki syndrome | 1 |
| Gastroenteritis | 2 | Atrioventricular block (AVB) | 1 |
| Moderate head injury | 2 | Sinus tachycardia | 1 |
| Minor head injury | 2 | Cardiopulmonary arrest | 1 |
| Cerebral palsy | 2 | | |
| Congenital hydrocephalus | 2 | Shock (unspecified) | 1 |
| Cerebral edema | 2 | Mitral regurgitation | 1 |
| Cerebral vascular accident | 2 | Cardiomyopathy | 1 |
| Convulsions in the newborn | 2 | Hepatic encephalopathy | 1 |
| Autoimmune disorder | 2 | Obstructive jaundice | 1 |
| Drug overdose | 2 | Neonatal jaundice | 1 |
| Leukemia | 2 | Failure to thrive | 1 |
| Sickle cell disease crisis | 2 | Tumor lysis syndrome | 1 |
| Acute kidney injury | 2 | Steven Johnson Syndrome | 1 |
| Prematurity | 2 | Acute viral bronchitis | 1 |
| Hypovolemic shock | 2 | Facial burns | 1 |
| Pulmonary embolism | 2 | | |

GIT, gastrointestinal tract; GUT, genitourinary tract; CNS, central nervous system.

**eTable 5. Detail of surgical procedures coded in the EPOK-Ped cohort**

| Type of procedure | Number of patients (n=109) | Type of procedure | Number of patients (n=109) |
|---|---|---|---|
| *Abdominal, GIT & GUT surgeries* | | Catheter insertion | 1 |
| Explorative laparotomy | 30 | Intravenous digital subtraction angiography (DSA) | 1 |
| Colectomy/Colostomy | 4 | *Other procedures* | |
| Ileal resection/ileostomy | 3 | Enucleation | 1 |
| Ileo-transverse anastomosis | 1 | Wound debridement | 4 |
| Pyloromyotomy | 4 | Suturing | 2 |
| Herniotomy | 1 | Removal of foreign body | 1 |
| Gastrotomy | 1 | Cleft lip repair | 1 |
| Manual reduction of intussusception | 2 | Palatoplasty | 1 |
| Repair of omphalocele | 2 | Partial nephrectomy | 1 |
| Repair of gastroschisis | 2 | Resection (unspecified) | 1 |
| Intestinal obstruction | 1 | | |
| Anoplasty | 1 | | |
| Orchidopexy | 3 | | |
| Urinary vesicostomy | 1 | | |
| Caesarean section/hysterotomy | 2 | | |
| *CNS Surgeries* | | | |
| Craniotomy | 10 | | |
| Primary hemilaminectomy for lumbar spine decompression | 1 | | |
| Repair of myelomeningocele | 1 | | |
| Ventricular-peritoneal shunt | 1 | | |
| Spinal fusion | 1 | | |
| *ENT Surgeries* | | | |
| Adenoidectomy | 3 | | |
| Tracheostomy | 1 | | |
| Excision of neck mass | 1 | | |
| Neck dissection | 1 | | |
| Bronchoscopy | 2 | | |
| Direct laryngoscopy with tumor excision | 1 | | |
| *Orthopedic/Bone procedures* | | | |
| Bone marrow aspiration | 1 | | |
| Correction of scoliosis | 1 | | |
| Open reduction of fracture | 1 | | |
| Percutaneous fixation | 1 | | |
| Pelvic osteotomy | 1 | | |
| Arthrotomy | 1 | | |
| *Cardiothoracic Surgery* | | | |
| Pulmonary lobectomy | 1 | | |
| Pulmonary segmentectomy | 1 | | |
| Thoracotomy (unspecified) | 1 | | |
| Insertion of chest tube | 3 | | |
| *Vascular surgeries* | | | |
| Cauterization | 1 | | |
| Exploration of artery of upper limb | 1 | | |

GIT, gastrointestinal tract; GUT, genitourinary tract; CNS, central nervous system

**eFigure 2. Duration of invasive mechanical ventilation in medical and surgical pediatric admissions, stratified by survival status**

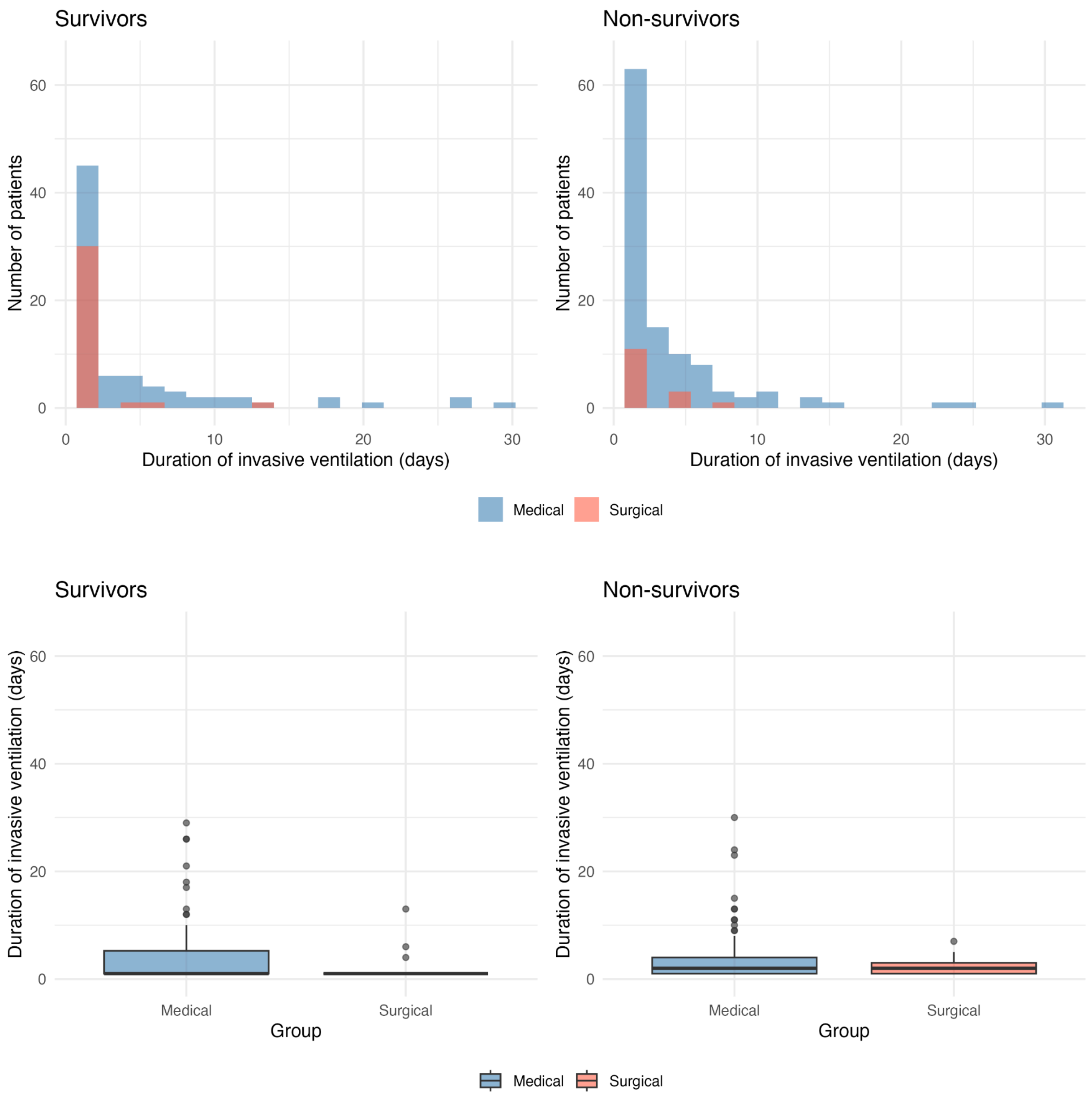

Supplement: Supplementary file 1 [file DataSheet1.pdf]
